# Nasopharyngeal Microbial Signatures Associated with Anti-VEGF Therapeutic Outcomes in Exudative Age-Related Macular Degeneration: A Pilot Study

**DOI:** 10.3390/ijms27219673

**Published:** 2026-10-28

**Authors:** Dzastina Cebatoriene, Akvile Bruzaite, Alvita Vilkeviciute-Petraite, Dalia Zaliuniene, Rokas Lukosevicius, Jurgita Skieceviciene, Juozas Kupcinskas, Rasa Liutkeviciene

**Affiliations:** 1 Medical Academy, Lithuanian University of Health Sciences, 44307 Kaunas, Lithuania; 2 Laboratory of Ophthalmology, Neuroscience Institute, Medical Academy, Lithuanian University of Health Sciences, 50161 Kaunas, Lithuania; 3 Department of Ophthalmology, Medical Academy, Lithuanian University of Health Sciences, 50161 Kaunas, Lithuania; 4 Laboratory of Clinical and Molecular Gastroenterology, Institute for Digestive Research, Lithuanian University of Health Sciences, 44307 Kaunas, Lithuania; 5 Department of Gastroenterology, Academy of Medicine, Lithuanian University of Health Sciences, 50161 Kaunas, Lithuania

**Keywords:** macular neovascularization, ranibizumab, bevacizumab, upper respiratory tract microbiome, 16S rRNA sequencing, *Corynebacterium*

## Abstract

Exudative age-related macular degeneration (AMD) is driven by choroidal angiogenesis, and the response to anti-vascular endothelial growth factor (anti-VEGF) therapy varies between patients. In this pilot study, we investigated whether the pretreatment nasopharyngeal microbiota differs between patients with exudative AMD and controls and whether it is associated with treatment response. Nasopharyngeal swabs were analysed by 16S rRNA gene (V3–V4) amplicon sequencing; after removal of host-derived, chloroplast and contaminant sequences and rarefaction, 55 samples (25 patients and 30 controls) were retained. Treatment response was assessed six months after starting therapy. Community composition was compared using permutational multivariate analysis of variance (PERMANOVA) adjusted for age, sex and statin use. Alpha and beta diversity did not differ between patients and controls (Shannon index, *p* = 0.892; PERMANOVA, *p* = 0.067). Pretreatment community composition differed between 19 responders and five non-responders (Bray–Curtis R^2^ = 0.057, *p* = 0.015; weighted UniFrac R^2^ = 0.143, *p* = 0.006), and non-responders had lower diversity (observed ASVs, *p* = 0.004). Non-responders had a higher relative abundance of *Corynebacterium* (median 71% vs. 19%; *p* = 0.009), which did not remain significant after correction for all genera tested. Pretreatment nasopharyngeal microbiota composition was associated with the response to anti-VEGF therapy but not with AMD status. These preliminary findings require confirmation in larger, independent cohorts.

## 1. Introduction

The human microbiota is the community of microorganisms—predominantly bacteria, together with fungi and viruses—that live in the human body. Due to its extensive genetic diversity and metabolic capabilities, the microbiota is increasingly seen as a functional organ that plays a key role in host physiology [1]. It is unevenly distributed across body parts, forming distinct microbial ecosystems on the skin, gastrointestinal tract, oral cavity, respiratory tract, urogenital tract, and ocular surface [2]. Microbiota dysbiosis—that is, changes in the quantity and quality of microbial composition and function—is marked by reduced diversity, overgrowth of opportunistic pathogens, and altered microbial metabolite production. Such changes can weaken epithelial barriers, increase mucosal permeability, and promote systemic low-grade inflammation through disrupted cytokine and chemokine signalling [3]. These mechanisms are now recognised as important contributors to the development of chronic inflammatory, metabolic, cardiovascular, and neurodegenerative diseases [3,4,5,6].

Chronic inflammatory conditions, such as inflammatory bowel diseases, coronary heart disease, arthritis, and peptic ulcer disease, are linked to changes in the composition of the gut and oral microbiota [3]. Alterations in microbial communities may contribute to mucosal inflammation and epithelial barrier dysfunction, potentially facilitating the systemic dissemination of microbial products and inflammatory mediators. These signals may subsequently influence immune responses in distant tissues and contribute to inflammatory processes [3,7]. Clinical and experimental evidence also suggests that signals from microbiota influence complement system activation and alter the behaviour of retinal microglia and macrophages, thereby impacting neovascularisation and neurodegeneration in the retina [8,9]. Age-related macular degeneration (AMD) is a complex, progressive neurodegenerative disease and a major cause of irreversible vision loss in older adults. In its exudative (neovascular) form, macular neovascularization—new vessels originating mainly from the choroid—leaks serous fluid, lipid and blood into and under the retina; on optical coherence tomography (OCT) these exudative changes appear as intraretinal fluid, subretinal fluid, pigment epithelial detachment and haemorrhage, and they cause rapid loss of central vision [10]. Vascular endothelial growth factor A (VEGF-A) is the principal driver of this angiogenesis, and intravitreal anti-VEGF therapy is the standard of care; nevertheless, a substantial proportion of patients show persistent or recurrent exudation despite treatment [11,12]. Its development involves oxidative stress, imbalance in the complement system, activation of pro-inflammatory cytokine pathways, and activation of microglial and macrophage cells. Genetic factors—especially variants in the complement factor H (*CFH*) and *ARMS2* genes—play a significant role in disease onset and progression [13]. Notably, CFH is a key regulator of the alternative complement pathway, and poor complement control is strongly linked to AMD development. Some microorganisms that can colonise silently, such as *Neisseria meningitidis* and *Streptococcus pneumoniae*, can recruit CFH to their surfaces to evade complement attack [14,15,16,17]. Previous studies have investigated potential interactions between CFH genetic variants and infectious agents, including *Chlamydia pneumoniae*, in relation to AMD susceptibility [13]. These observations suggest a possible relationship between microbial exposure, complement regulation, and AMD pathogenesis.

Although most research has focused on the gastrointestinal microbiota, increasing attention is being paid to microbial niches outside the gut, such as the ocular surface and the upper respiratory tract. The ocular microbiota, despite its low abundance and diversity—partly due to continuous tear flow and antimicrobial components—plays a role in local immune regulation, resistance to colonisation, and the maintenance of epithelial barriers [4,5,18,19]. Recent studies have explored the ocular microbiota in inflammatory and degenerative eye diseases, including AMD, highlighting the importance of the eye–gut–immune axis in controlling systemic and local inflammation [7,9,19,20,21]. The nasopharynx harbours a distinct microbial community that contributes to mucosal immune homeostasis. In healthy adults, the nose and throat typically harbour bacteria from genera such as *Streptococcus*, *Haemophilus*, *Neisseria*, *Moraxella*, *Corynebacterium*, and *Staphylococcus*, with their composition influenced by age, smoking, infections, antibiotic use, and environmental factors [22,23,24,25]. The ocular surface and nasal cavity are anatomically connected through the nasolacrimal drainage system, which provides a potential route for interactions between their respective mucosal environments. Studies investigating the microbiota of the nasolacrimal system have identified microbial communities that may share characteristics with those of adjacent ocular and nasal niches [26,27,28]. These compartments are functionally linked by shared mucosal immunity supported by mucosa-associated lymphoid tissues, including nasal-associated lymphoid tissue (NALT) and lacrimal drainage-associated lymphoid tissue (LDALT) [29,30].

Dysbiosis of the nasopharyngeal microbiota has been linked to conditions such as mucosal barrier dysfunction, chronic rhinosinusitis, recurrent respiratory infections, and increased local and systemic inflammation [22]. Because of the anatomical and immune-system connections between the nasopharynx and the ocular surface, changes in nasopharyngeal microbial composition may affect ocular immune balance [30]. By influencing secretory IgA responses, complement activity, cytokine levels, and epithelial barriers, nasopharyngeal dysbiosis might foster an inflammatory environment that could impact retinal health [3,22].

Emerging case–control studies suggest a possible link between microbiota composition and AMD. Changes in gut microbial profiles have been observed in patients with neovascular AMD [31], and differences in pharyngeal microbiota—such as higher levels of *Gemella* and lower levels of *Prevotella*—have been observed in AMD groups compared to controls [32]. Rullo et al. [8] reported differences in nasal and oral microbial communities between patients with neovascular AMD and controls. Although these findings suggest a potential association between extraocular microbial composition and AMD, the underlying inflammatory mechanisms remain to be established.

Collectively, existing evidence suggests that alterations in microbial communities may influence inflammatory and immune-regulatory pathways implicated in AMD pathogenesis. However, the relationship between nasopharyngeal microbiota composition, neovascular AMD, and response to anti-vascular endothelial growth factor (anti-VEGF) therapy remains poorly understood. Given the involvement of complement activation, cytokine signalling, and immune-cell activity in retinal inflammation and angiogenesis, investigating nasopharyngeal microbial communities may provide further insight into potential host–microbiota interactions associated with therapeutic outcomes.

To our knowledge, no previous study has examined whether the pretreatment nasopharyngeal microbiota is associated with the response to anti-VEGF therapy, which is the novel aspect of this work. Therefore, this pilot study aimed to characterise the nasopharyngeal microbiota in patients with neovascular AMD and healthy controls and to investigate whether microbial community composition and specific bacterial taxa are associated with response to anti-VEGF therapy. We hypothesised that differences in nasopharyngeal microbial composition may be associated with therapeutic response, potentially reflecting underlying host–microbiota interactions relevant to inflammatory and angiogenic processes.

## 2. Results

### 2.1. Characteristics of the Study Participants

**Figure 1:**
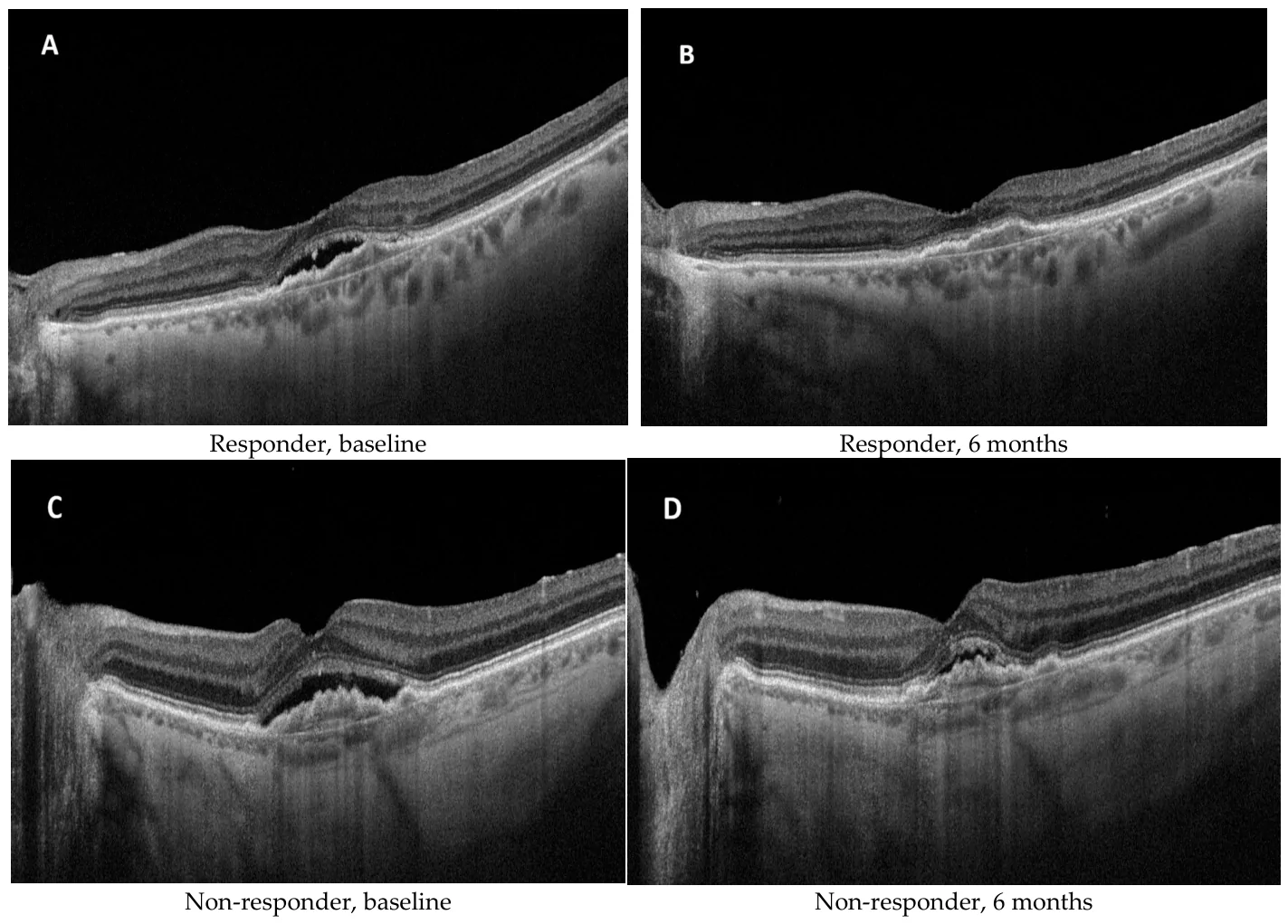
Representative OCT B-scans of a responder ((**A**), baseline; (**B**), six months) and a non-responder ((**C**), baseline; (**D**), six months).

The demographic and clinical characteristics of the participants are summarised in Table 1. Patients with AMD were older than controls (median 80 (IQR 73–84) vs. 69.5 (IQR 58–76) years, *p* = 0.001) and more often used statins (16/24 vs. 6/30, *p* < 0.001), whereas the sex distribution was similar (76% vs. 80% women, *p* = 0.75). After six months of treatment, median CMT in patients with AMD decreased from 381 (IQR 327–438) to 300 (IQR 275–346) µm (Wilcoxon signed-rank *p* = 0.001). In responders, BCVA improved (median change −0.09 logMAR, IQR −0.21 to −0.02; *p* < 0.001), whereas four of the five non-responders lost visual acuity (increase of 0.15–0.93 logMAR) and one had an increase in CMT of 124 µm with stable acuity, consistent with the response criteria. Representative OCT B-scans illustrating anatomical changes from baseline to six months in a responder and a non-responder are presented in Figure 1. Responders and non-responders did not differ at baseline in age, sex, statin use, BCVA or CMT (Table 1). One patient did not attend the six-month visit, so treatment response could not be assessed.

### 2.2. Study Cohort and Sequencing Depth

**Figure 2:**
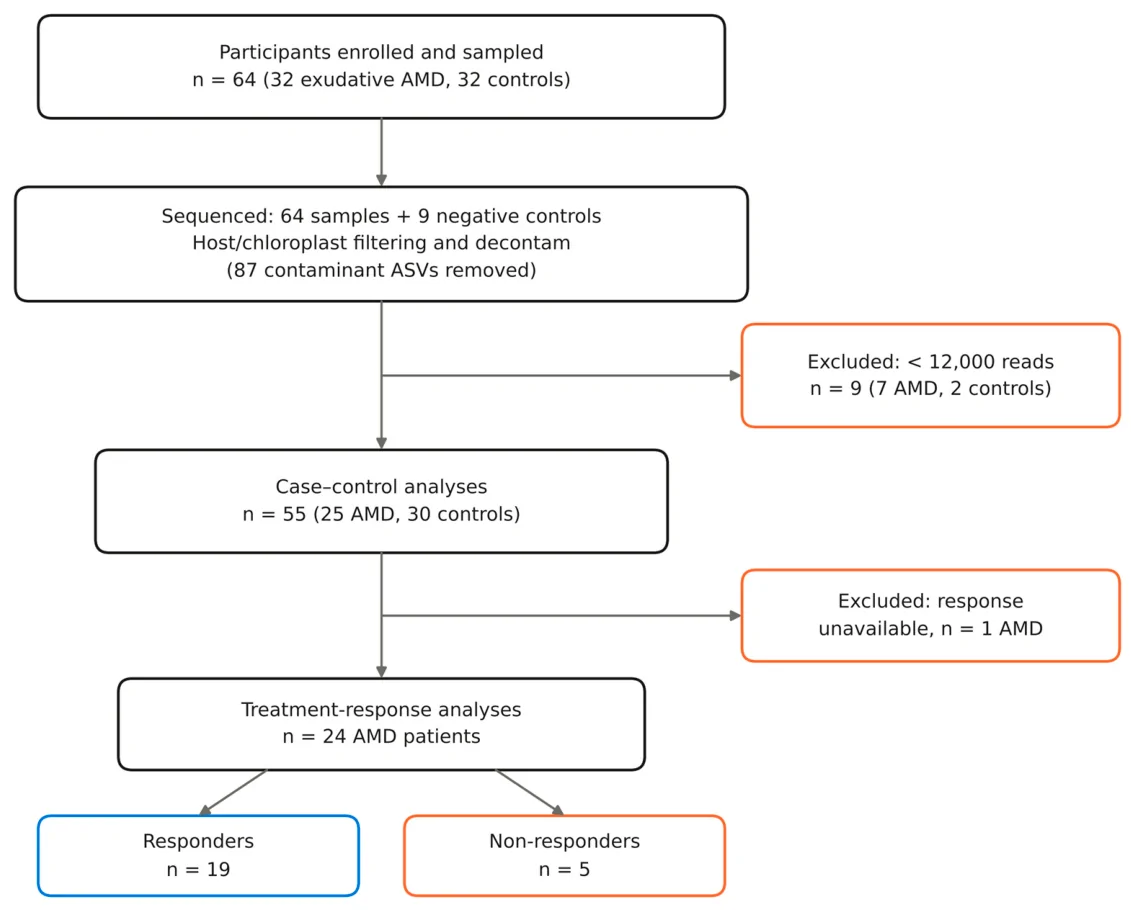
Flow of participants and samples through the study. Samples were excluded if fewer than 12,000 reads remained after removal of host-derived, chloroplast and contaminant sequences.

A total of 73 sequencing libraries were generated, including nine negative controls (extraction blanks and PCR controls). After removal of control libraries, 64 human samples remained, comprising 32 patients with neovascular age-related macular degeneration (AMD) and 32 control subjects. The flow of participants and samples is shown in Figure 2.

Mitochondrial (host-derived) and chloroplast sequences and ASVs without a bacterial phylum assignment (1732 ASVs, 5.9% of reads from participant samples) were removed. The decontam procedure identified 87 contaminant ASVs (2.2% of the remaining reads), dominated by typical reagent-associated taxa such as *Burkholderia–Caballeronia–Paraburkholderia* (Supplementary Table S1). Extraction and swab blanks also contained low numbers of reads assigned to genera abundant in the samples, including *Corynebacterium* (4–16% of blank reads), consistent with low-level cross-contamination; the dominant *Corynebacterium* ASVs were not classified as contaminants.

After depth filtering, 55 samples remained for analysis, including 30 control subjects and 25 AMD patients (Figure 2). Among the AMD patients retained after rarefaction, treatment response information was unavailable for one individual. After excluding this sample, 24 AMD patients were included in treatment-response analyses, comprising 19 responders and 5 non-responders.

Across all retained samples, 13,705 amplicon sequence variants (ASVs) were detected after denoising, quality filtering and decontamination. After rarefaction to 12,000 reads per sample, the final dataset contained 660,000 reads across 55 samples.

### 2.3. Alpha Diversity in AMD Patients and Controls

Alpha-diversity analyses were performed on the 55 retained samples (30 controls and 25 AMD patients).

**Figure 3:**
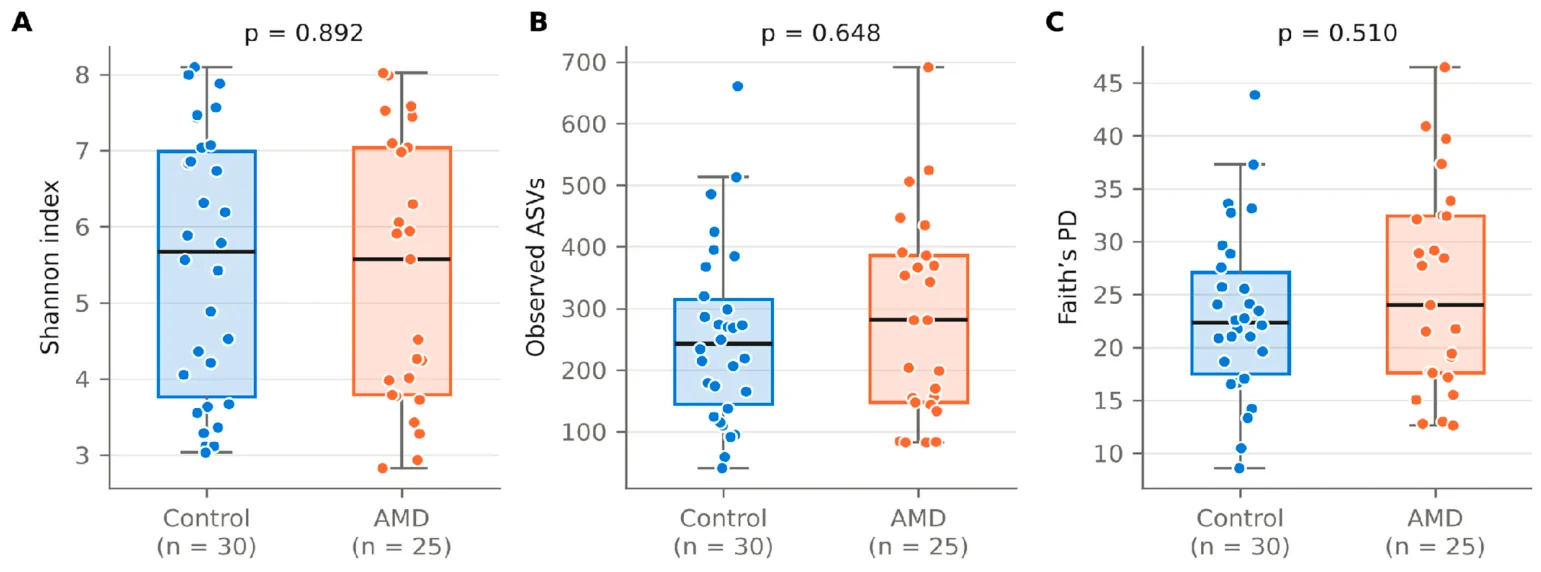
Alpha diversity of the nasopharyngeal microbiome in AMD patients and controls. Boxplots show the Shannon diversity index (**A**), observed ASVs (**B**) and Faith’s phylogenetic diversity (**C**) after rarefaction to 12,000 reads per sample; each point represents one participant. *p*-values from the Kruskal–Wallis test.

No significant differences in within-sample microbial diversity were observed between AMD patients and controls. The Shannon diversity index was comparable between groups (Kruskal–Wallis χ^2^ = 0.02, *p* = 0.892). Similarly, observed features, reflecting species richness, did not differ significantly between AMD patients and controls (χ^2^ = 0.21, *p* = 0.648). Faith’s phylogenetic diversity was also comparable between groups (χ^2^ = 0.43, *p* = 0.510) (Figure 3).

Together, these findings indicate similar microbial richness and overall diversity between AMD patients and controls.

### 2.4. Beta Diversity—Case–Control Comparison

Beta-diversity analysis was performed to evaluate differences in microbial community composition between AMD patients and controls.

Initial analyses using Bray–Curtis dissimilarity did not show clear separation between groups. To control for potential confounding factors, a multivariable PERMANOVA model was applied, including age, sex, and statin use. After adjustment, disease status explained 2.1% of the variation in community composition, which was not statistically significant (R^2^ = 0.021, *p* = 0.067); the full model including covariates explained 8.1%.

Similarly, phylogeny-aware beta-diversity metrics showed no significant differences between groups. Neither weighted UniFrac distance (R^2^ = 0.014, *p* = 0.598) nor unweighted UniFrac distance (R^2^ = 0.018, *p* = 0.361) demonstrated significant group separation.

**Figure 4:**
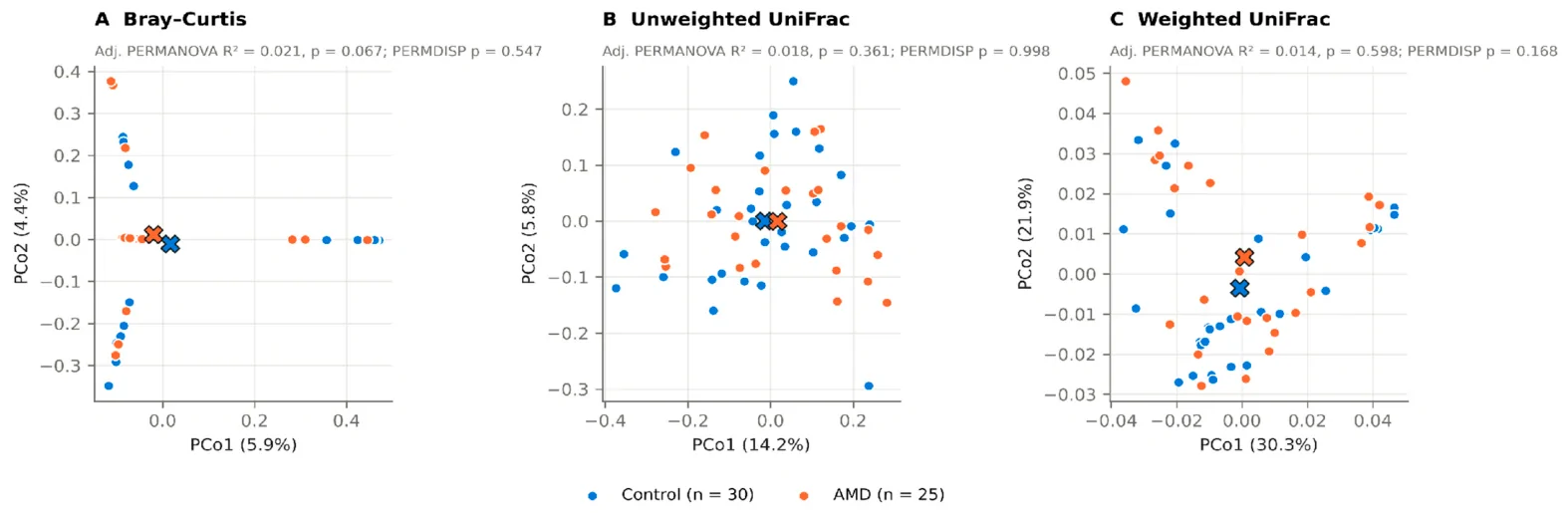
Beta diversity of the nasopharyngeal microbiome in AMD patients and controls. Principal coordinate analysis (PCoA) based on Bray–Curtis (**A**), unweighted UniFrac (**B**) and weighted UniFrac (**C**) distances. Crosses mark group centroids. PERMANOVA adjusted for age, sex and statin use; PERMDISP tests homogeneity of dispersion.

Homogeneity of multivariate dispersion was confirmed using PERMDISP (Bray–Curtis *p* = 0.55; unweighted UniFrac *p* = 0.998; weighted UniFrac *p* = 0.17), indicating that PERMANOVA results were not driven by unequal within-group variability (Figure 4). Results were unchanged after exclusion of four controls younger than 50 years (adjusted Bray–Curtis *p* = 0.062).

Together with the alpha-diversity results, these findings indicate that exudative AMD is not associated with large-scale alterations in the nasopharyngeal microbial community structure.

### 2.5. Differential Abundance—Case–Control Comparison

Genus-level differential abundance analysis was conducted on CLR-transformed counts with prevalence filtering (10%) and false discovery rate (FDR) correction.

**Figure 5:**
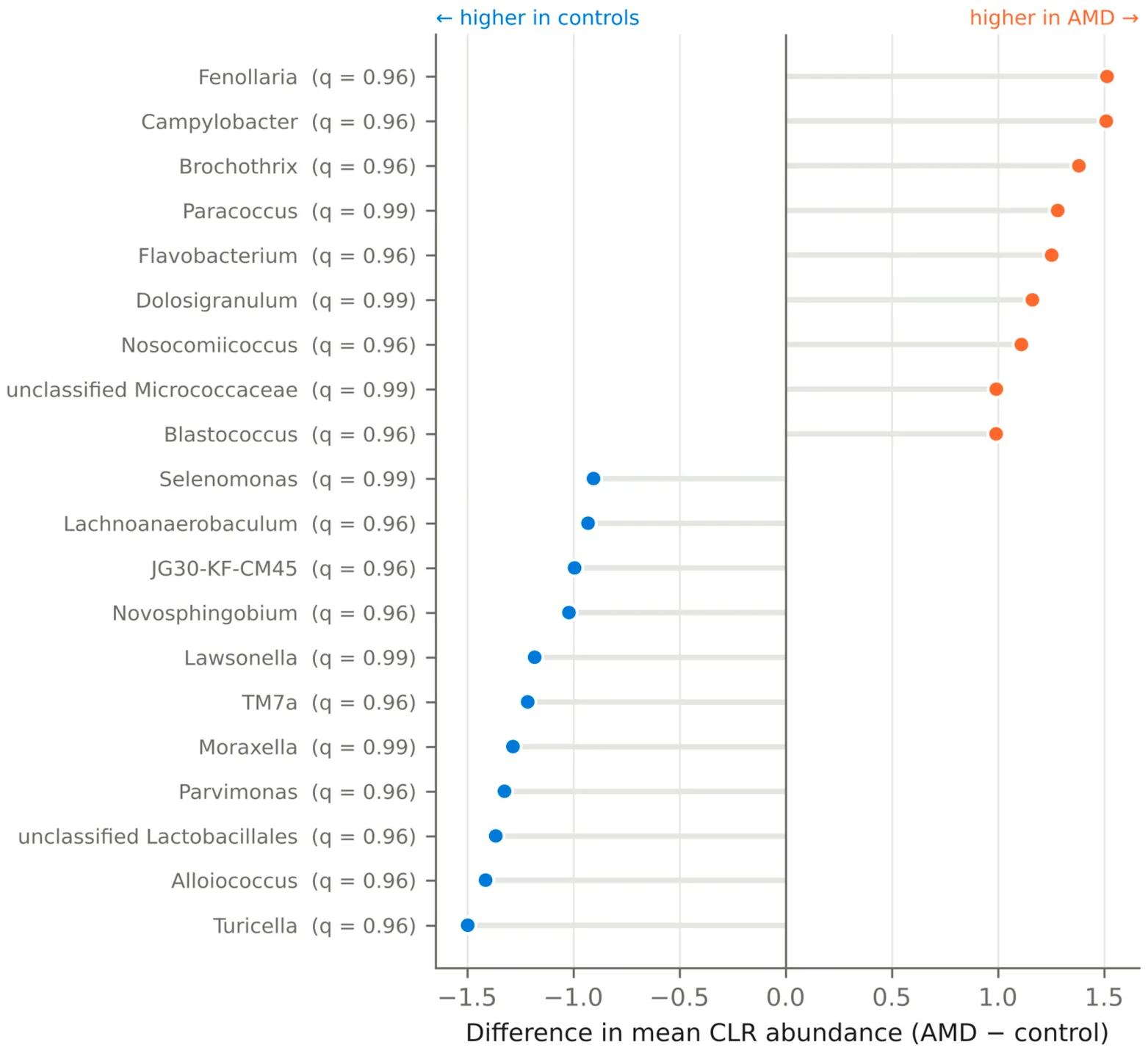
Genus-level differential abundance between AMD patients and controls. The 20 genera with the largest effect sizes are shown, with FDR-adjusted q-values. No genus reached statistical significance after multiple-testing correction.

No bacterial genera showed a significant association with AMD status after correction for multiple testing (all FDR-adjusted q-values > 0.05) (Figure 5). The same was true with a 5% prevalence threshold.

Although several taxa showed nominal associations before FDR adjustment, none remained statistically significant after correcting for multiple comparisons.

Figure 5 shows the genera with the largest estimated effect sizes.

These findings further support the observation that AMD status alone does not drive reproducible compositional shifts in the nasopharyngeal microbiome.

### 2.6. Treatment Response-Associated Microbiome Differences

#### Community-Level Compositional Differences

To determine whether microbial composition was associated with therapeutic response, beta-diversity analyses were performed within the AMD patient cohort.

This analysis included 19 responders and 5 non-responders.

Non-responders had lower alpha diversity than responders (observed ASVs, median 134 vs. 353, Kruskal–Wallis *p* = 0.004; Faith’s PD, 15.5 vs. 28.9, *p* = 0.003; Shannon index, 3.7 vs. 6.1, *p* = 0.012).

Significant separation between responders and non-responders was observed using Bray–Curtis dissimilarities, with PERMANOVA indicating a statistically significant difference between groups (pseudo-F = 1.31, *p* = 0.045). A similar result was observed using unweighted UniFrac distance, which also showed significant group separation (pseudo-F = 1.85, *p* = 0.007).

Weighted UniFrac distance, which accounts for the relative abundance of dominant taxa, also differed between response groups (pseudo-F = 3.56, *p* = 0.005).

**Figure 6:**
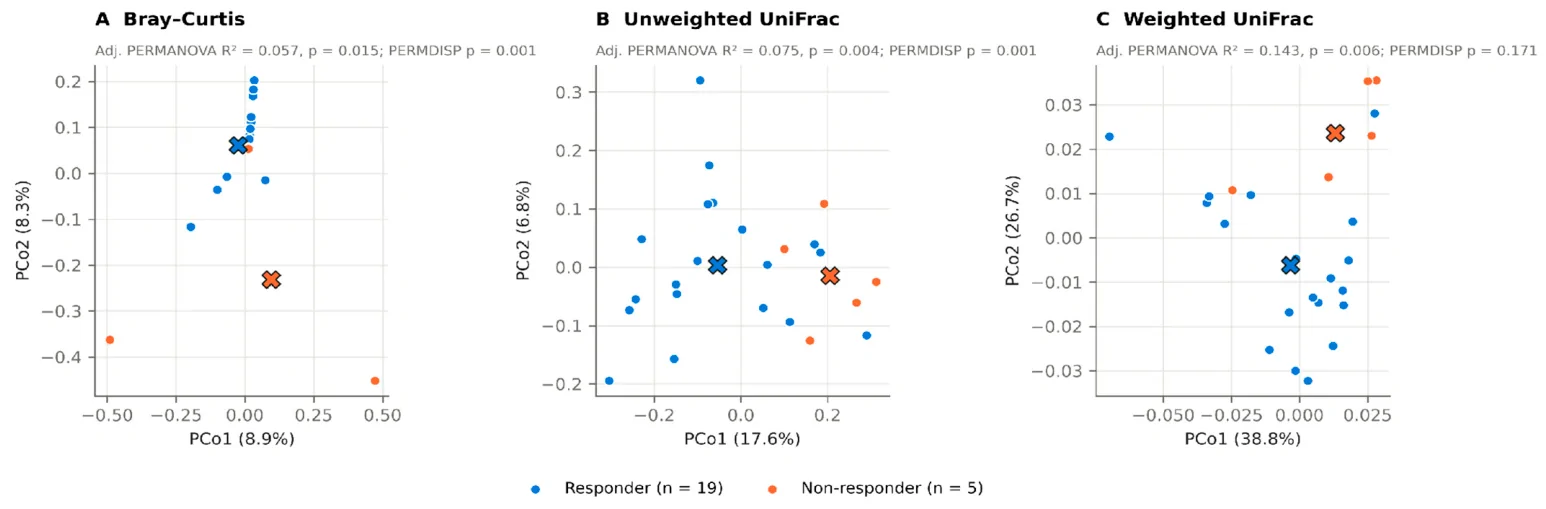
Principal coordinate analysis (PCoA) of nasopharyngeal microbiome composition according to treatment response: Bray–Curtis (**A**), unweighted UniFrac (**B**) and weighted UniFrac (**C**) distances. Crosses mark group centroids. PERMANOVA adjusted for age, sex and statin use.

These findings indicate that treatment response was associated with differences in both community membership and the relative abundance of dominant taxa (Figure 6).

### 2.7. Covariate-Adjusted Response Analysis

To account for potential confounding factors, a multivariable PERMANOVA model including age, sex, and statin use was fitted.

Treatment response remained significantly associated with microbial community composition after covariate adjustment, with response explaining 5.7% of the variation (Bray–Curtis R^2^ = 0.057, *p* = 0.015; unweighted UniFrac R^2^ = 0.075, *p* = 0.004; weighted UniFrac R^2^ = 0.143, *p* = 0.006; *n* = 23 with complete covariate data).

Further adjustment for baseline BCVA, baseline CMT or the anti-VEGF agent, entered one at a time, did not change these findings (Bray–Curtis *p* = 0.008, *p* = 0.001 and *p* = 0.008; unweighted UniFrac *p* = 0.001, *p* = 0.002 and *p* = 0.002; weighted UniFrac *p* < 0.001, *p* < 0.001 and *p* = 0.002).

Multivariate dispersion differed between response groups for Bray–Curtis and unweighted UniFrac distances (PERMDISP *p* = 0.001), with non-responders being more homogeneous than responders, but not for weighted UniFrac (*p* = 0.17). Because the smaller group had the smaller dispersion, PERMANOVA is conservative in this setting [33], and the weighted UniFrac result, which was not accompanied by a dispersion difference, supports a true difference in community location.

These associations were robust to the rarefaction draw (adjusted Bray–Curtis *p* = 0.008–0.020 across 20 rarefactions), to a lower rarefaction depth of 9000 reads (*n* = 60; *p* = 0.013) and to the decontamination threshold. In leave-one-out analyses, the weighted UniFrac association remained significant after removal of each non-responder (*p* = 0.001–0.050), whereas the Bray–Curtis association lost significance when either of two non-responders was removed (*p* = 0.21 and *p* = 0.38) (Supplementary Table S2).

These results indicate that baseline microbial community composition differed according to the subsequent treatment outcome.

### 2.8. Taxon-Level Association with Treatment Response

To identify taxa contributing to response-associated compositional differences, genus-level abundances were compared between responders and non-responders.

**Figure 7:**
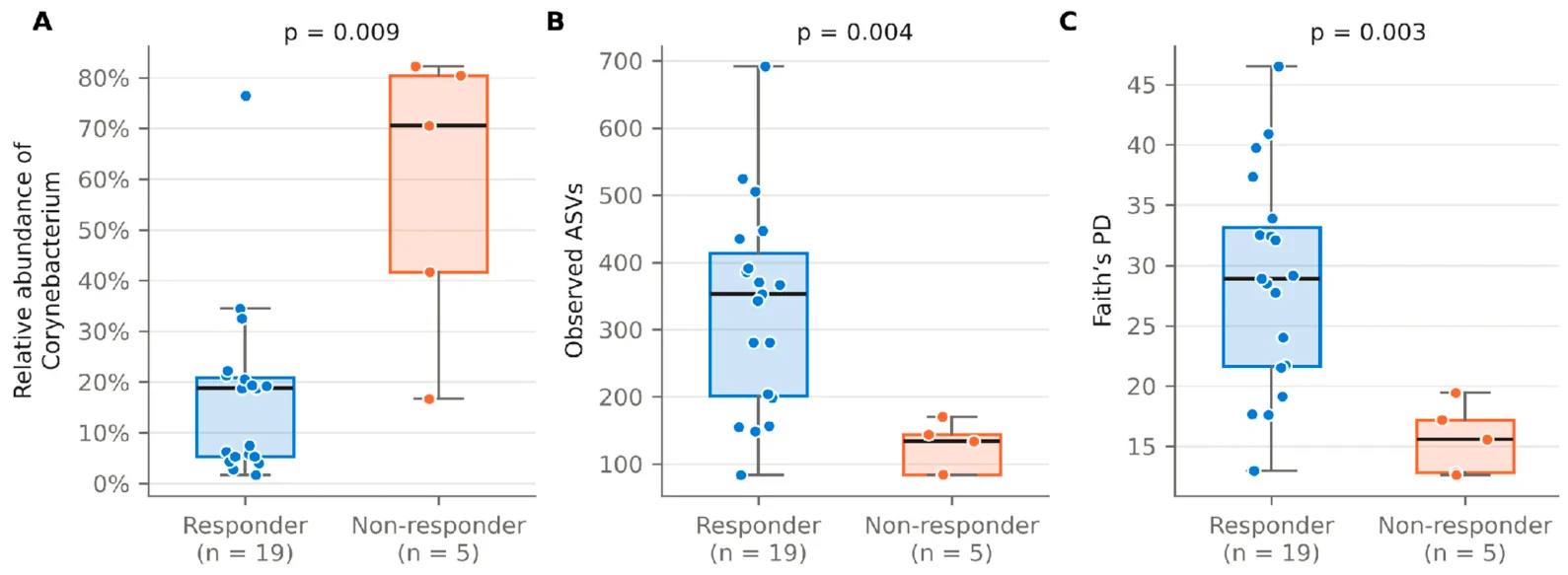
*Corynebacterium* relative abundance (**A**), observed ASVs (**B**) and Faith’s phylogenetic diversity (**C**) according to treatment response. Each point represents one patient; boxplots show the median and interquartile range. *p*-values from the Wilcoxon rank-sum (**A**) and Kruskal–Wallis (**B**,**C**) tests.

In a post hoc exploratory comparison, the genus *Corynebacterium* showed the largest difference between response groups. The relative abundance of *Corynebacterium* was significantly higher in non-responders compared with responders (median 70.5%, IQR 41.7–80.4, vs. 18.7%, IQR 5.3–20.9; Wilcoxon rank-sum test, *p* = 0.009) (Figure 7A; alpha diversity by response group is shown in Figure 7B,C).

Effect size analysis confirmed a large difference between groups, with Cliff’s delta = 0.75 (95% bootstrap CI 0.28–1.00). The difference persisted in all leave-one-out analyses (*p* = 0.001–0.035) and in all 20 rarefactions. However, in a genus-wide comparison of 350 genera, *Corynebacterium* ranked first but did not remain significant after FDR correction (q = 0.12; Supplementary Figure S1).

Importantly, *Corynebacterium* was detected in all samples, indicating that the observed association reflects quantitative expansion of this taxon rather than differences in colonisation frequency.

### 2.9. Genus-Level Compositional Patterns

**Figure 8:**
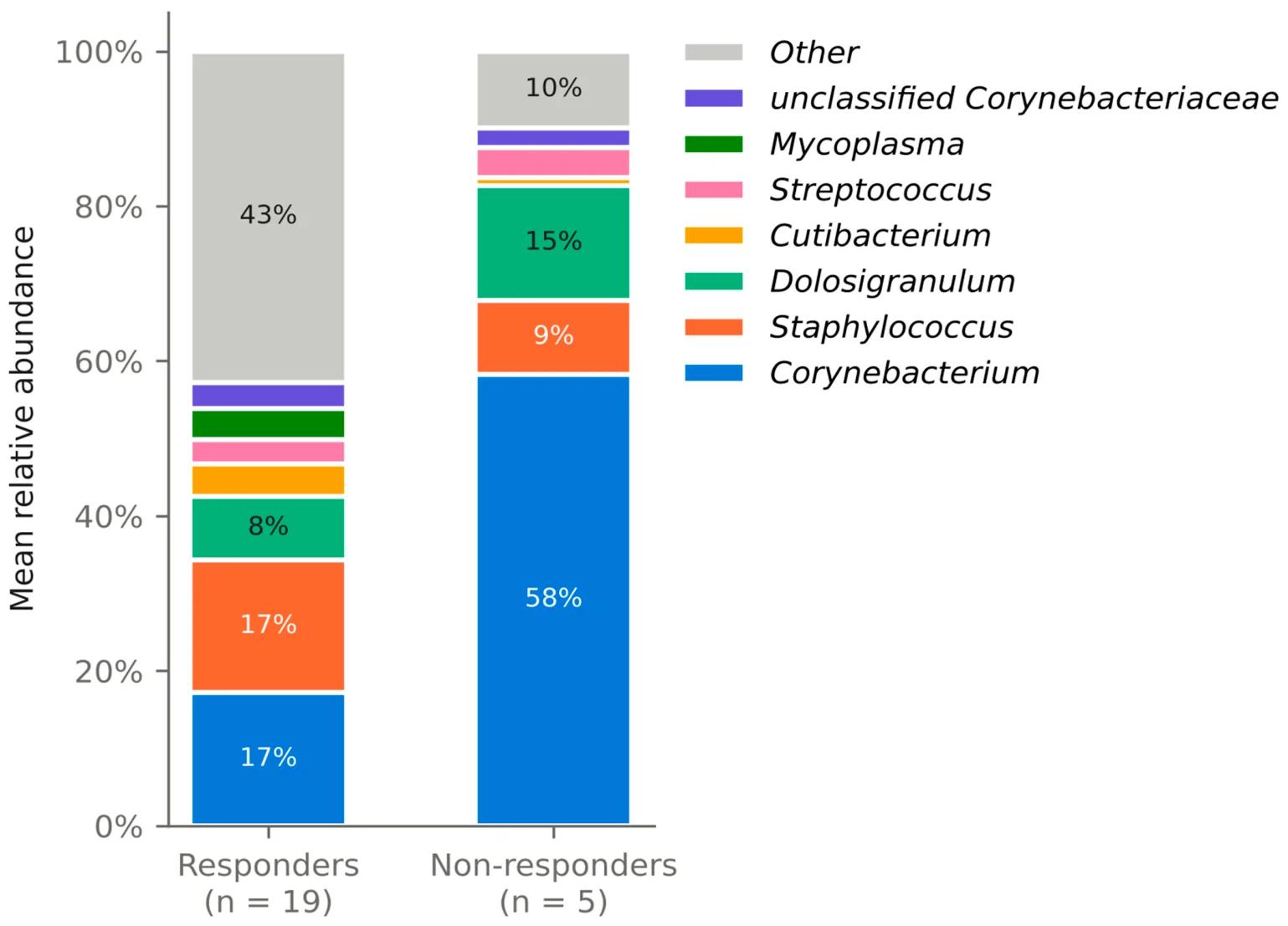
Genus-level composition by treatment response. Stacked bars show the mean per-sample relative abundances of the seven most abundant genera in responders (*n* = 19) and non-responders (*n* = 5); remaining genera are grouped as “Other”.

Genus-level compositional profiles further illustrated differences between treatment-response groups. Non-responders exhibited microbiomes dominated by *Corynebacterium*, whereas responders showed a more heterogeneous microbial composition with relatively higher representation of anaerobic genera such as *Cutibacterium* and *Anaerococcus* (non-responders vs. responders: *Corynebacterium*, mean 58% vs. 17% of reads; *Cutibacterium*, 1.0% vs. 4.2%; *Anaerococcus*, 0.7% vs. 2.5%) (Figure 8).

Although these compositional differences are descriptive, they are consistent with response-associated differences in baseline community composition within the AMD cohort.

## 3. Discussion

In this case–control study, we investigated whether exudative age-related macular degeneration (AMD) is associated with alterations in nasopharyngeal microbiota composition and whether microbial community structure is linked to response to anti-VEGF therapy. Overall, our findings indicate that exudative AMD itself is not associated with marked, community-wide alterations in the nasopharyngeal microbiota. In contrast, treatment response was associated with differences in microbial community structure, suggesting a potential modulatory role of the microbiota in therapeutic outcomes rather than in the development of primary disease.

### 3.1. Nasopharyngeal Microbiota in Exudative AMD: Absence of Global Dysbiosis

In our cohort, neither alpha diversity, beta diversity nor genus-level abundance differed between patients with exudative AMD and controls, suggesting that a consistent, large-scale restructuring of the nasopharyngeal microbial community does not characterise exudative AMD. If microbiota-related mechanisms contribute to disease pathogenesis, they are unlikely to manifest as global dysbiosis in this anatomical niche. Instead, any disease-associated signals are likely subtle, taxonomically selective, or functionally mediated rather than reflected by broad compositional shifts.

Previous studies have reported associations between upper respiratory tract microbiota and AMD. Ho et al. [32], analysing the 16S rRNA gene V3–V6 region using the Illumina MiSeq platform, described genus-level differences in a large cohort and identified candidate genera such as *Prevotella* and *Gemella* that may be associated with AMD development and progression. However, in contrast to the findings reported by Ho et al., our analysis did not reveal statistically significant differences in overall nasopharyngeal microbiota diversity or community structure between patients with exudative AMD and controls. This discrepancy may be explained by methodological differences between studies, including cohort size, anatomical sampling site (pharyngeal versus nasopharyngeal microbiota), targeted 16S rRNA regions (V3–V6 versus V3–V4), and analytical pipelines used for sequence processing and taxonomic assignment. Instead, host–microbiota interactions may influence disease-related pathways without necessarily inducing large-scale restructuring of microbial communities.

Gut microbiome studies have produced more consistent signals. Zinkernagel et al. [31] reported enrichment of *Anaeroglobus*, *Oscillibacter* and *Ruminococcus torques* in patients with neovascular AMD, and Zysset–Burri et al. [34] linked intestinal taxa to systemic complement activation in neovascular AMD. Together with our findings, this suggests that, if extraocular microbiota contribute to AMD, the gut may be a more relevant niche than the upper airway, or that airway-related effects act through host immune tone rather than through overall community structure.

**Microbiota structure and response to anti-VEGF therapy**

In contrast to disease status, treatment responses demonstrated a clearer association with microbiota structure. Beta diversity analysis restricted to the patient cohort revealed statistically significant differences between responders and non-responders using Bray–Curtis, unweighted and weighted UniFrac distances, and the association persisted after adjustment for age, sex and statin use, with response explaining 6–14% of community variation depending on the metric. Non-responders also had markedly lower alpha diversity and more homogeneous communities, consistent with dominance by a single genus.

At the genus level, *Corynebacterium* showed the strongest nominal association with treatment response. Although no genus remained significant after false discovery rate correction in the global comparison, post hoc analysis of this taxon demonstrated significantly higher relative abundance in non-responders (Wilcoxon *p* = 0.009), with a large effect size (Cliff’s δ = 0.75). *Corynebacterium* was detected in all samples, indicating that the association reflects quantitative enrichment rather than differential colonisation.

Similarly, Rullo et al. [8] reported significant differences in oral and nasal microbiota between AMD patients and controls, including enrichment of Actinobacteriota-related taxa in affected individuals. In their case–control study, the nasal and oral microbiota of 13 patients with newly diagnosed neovascular AMD (6 males, 7 females) were compared with that of 5 control subjects without retinal diseases (2 males, 3 females). As *Corynebacterium* belongs to the Actinobacteriota phylum, the increased relative abundance of this genus observed in non-responders in our study is partly consistent with their findings. To our knowledge, no studies have yet evaluated the association between nasopharyngeal microbiota and AMD in the context of response to anti-VEGF therapy.

*Corynebacterium* are generally regarded as commensal members of the upper respiratory tract microbiota that contribute to mucosal barrier integrity and immune homeostasis [35]. In the anterior nares, *Corynebacterium accolens* inhibits *Streptococcus pneumoniae* by releasing free fatty acids from host lipids [36], and *Corynebacterium*/*Dolosigranulum*-dominated upper airway profiles have been associated with respiratory health [24]. However, certain representatives have been increasingly recognised as opportunistic pathogens [37], and *Corynebacterium* have been reported as potential causes of pneumonia in mechanically ventilated patients [38]. Whether the expansion of this genus in non-responders reflects a harmful process, a protective response or simply a marker of an altered mucosal environment, epithelial barrier dysfunction or subclinical inflammation cannot be determined from our data.

In contrast, responders demonstrated a more diverse and heterogeneous genus-level composition, with relatively greater representation of anaerobic genera such as *Cutibacterium* and *Anaerococcus*. Although the functional role of these taxa in AMD remains unclear, greater microbial diversity is generally associated with increased ecological resilience and broader functional capacity within microbial communities [22].

**Interpretation and clinical implications**

Anti-VEGF agents such as ranibizumab and bevacizumab bind VEGF-A and thereby suppress endothelial cell proliferation, vascular permeability and neovascular growth, leading to resolution of exudation and stabilisation or improvement in vision in most patients; the two agents have comparable efficacy [12,39]. Inadequate response has been attributed to lesion characteristics, tachyphylaxis and activation of VEGF-independent inflammatory and angiogenic pathways [11,12]. A host inflammatory state shaped in part by mucosal microbiota could plausibly contribute to such VEGF-independent activity.

Growing evidence suggests that the respiratory microbiota can influence host immune signalling through interactions with epithelial and innate immune cells at mucosal surfaces. Microbial communities in the upper respiratory tract play a crucial role in controlling local immune balance and inflammatory signalling networks [22]. Experimental studies have demonstrated that commensal bacteria can activate innate immune pathways and trigger the production of cytokines such as IL-6, TNF-α, and interferon-related mediators [40,41]. These mediators are known to interact with inflammatory and angiogenic signalling pathways involved in the pathogenesis of AMD, suggesting that microbiota-driven immune modulation could influence retinal vascular responses to anti-VEGF therapy. Although the nasopharyngeal microbiome is anatomically distant from the retina, mucosal–systemic immune crosstalk presents a plausible mechanism linking airway microbial composition with systemic inflammatory tone [3]. In this context, enrichment of *Corynebacterium* in non-responders may indicate an underlying host inflammatory state that affects the response to anti-VEGF therapy, rather than a direct causal role of the bacterium itself. Importantly, the relatively small number of non-responders in this cohort warrants cautious interpretation of taxon-level findings. The differences observed regarding treatment response were based on relative abundance data and do not establish causality. Therefore, these findings should be considered preliminary and serve as hypotheses to be tested.

In summary, exudative AMD was not associated with pronounced, community-wide alterations in the nasopharyngeal microbiota. However, specific compositional features—particularly enrichment of *Corynebacterium*—were associated with response to anti-VEGF therapy. These results support the concept that the nasopharyngeal microbiota may be associated with disease-related processes and contribute to variability in therapeutic response, rather than serving as a primary driver of disease onset.

Larger prospective studies integrating microbiome profiling with functional and immunological analyses will be necessary to validate these associations and determine their biological relevance. If confirmed in independent cohorts, microbiome-derived signatures could contribute to precision medicine by identifying patients at risk of a suboptimal response to anti-VEGF therapy. Taken together, these findings suggest that host–microbiome interactions may contribute to therapeutic heterogeneity in exudative AMD.

Based on our data, and assuming a more conservative effect size than observed (Cliff’s δ = 0.5) with a similar proportion of non-responders (21%), approximately 64 patients (about 13 non-responders) would be required to detect a difference in *Corynebacterium* abundance with 80% power at α = 0.05; for a small-to-moderate effect (δ = 0.33), about 146 patients (30 non-responders) would be needed [42].

### 3.2. Study Limitations

Several limitations should be considered when interpreting the present findings. First, this was a pilot study without an a priori sample size calculation, and it included a relatively small cohort, particularly in the treatment-response analysis, which comprised 19 responders and only five non-responders. This substantial imbalance limits statistical power and the precision of effect estimates and warrants caution when interpreting the multivariable analyses and observed differences in microbial community composition. In leave-one-out analyses, the Bray–Curtis association depended on two non-responders, although the weighted UniFrac and *Corynebacterium* findings were stable.

Second, although nasopharyngeal samples were collected before the initiation of anti-VEGF therapy, microbiome composition was assessed at a single time point. Consequently, potential changes in microbial communities during treatment could not be evaluated. Furthermore, the observational study design does not allow causal relationships between baseline microbial composition and subsequent treatment response to be established.

Third, although the multivariable analyses adjusted for age, sex, and statin use, other potentially relevant factors were not accounted for. Patients were also older and more often used statins than controls; although both variables were included as covariates, residual confounding by age cannot be excluded. Smoking status, alcohol consumption and recent antibiotic or glucocorticoid use were not recorded, although smoking in particular is known to alter the upper airway microbiota [43]; their potential confounding effects could therefore not be assessed. In addition, the number of anti-VEGF injections and the treatment intervals were not available for analysis, and, owing to the small sample, the influence of the anti-VEGF agent and of baseline BCVA and CMT could only be explored in sensitivity analyses, in which none of them altered the results. Residual confounding therefore cannot be excluded.

Fourth, the association involving *Corynebacterium* was identified through a post hoc exploratory analysis performed after inspection of the microbial composition results. Although its relative abundance was higher in non-responders, the reported Wilcoxon *p*-value was not accompanied by a multiple-testing adjustment for this specific comparison. Given the exploratory nature of the analysis and the small number of non-responders, this finding should be considered hypothesis-generating rather than evidence of a validated microbial biomarker of treatment response.

Fifth, the use of 16S rRNA gene sequencing limits taxonomic resolution and does not directly establish microbial functional activity. Furthermore, the relative abundance data do not allow changes in absolute bacterial abundance to be determined. Inflammatory mediators, complement activity, and immune-cell responses were not directly measured. Accordingly, the proposed involvement of inflammatory signalling in the relationship between nasopharyngeal microbial composition and anti-VEGF treatment response remains hypothetical.

Sixth, extraction and swab blanks contained reads from genera abundant in the samples, including *Corynebacterium*, most likely reflecting low-level cross-contamination during processing. Although statistical decontamination was applied and the dominant *Corynebacterium* variants were not identified as contaminants, a minor contribution of cross-contamination to the measured abundances cannot be fully excluded.

Finally, the findings were not validated in an independent cohort, and the predictive performance of baseline microbial composition was not formally evaluated.

## 4. Methods

### 4.1. Establishment of the Study Cohorts

This single-centre, prospective case–control pilot study was conducted at the Department of Ophthalmology, Hospital of the Lithuanian University of Health Sciences Kauno Klinikos, between February 2025 and February 2026. It is reported following the STROBE statement and the STORMS checklist for human microbiome studies [44,45]. The flow of participants and samples through the study is shown in Figure 2. The study was carried out in accordance with the principles of the Declaration of Helsinki. Ethical approval was obtained from the Kaunas Regional Biomedical Research Ethics Committee of the Lithuanian University of Health Sciences (approval No. BE-2-/48).

All participants underwent a comprehensive ophthalmological examination, during which information regarding their overall health status and comorbid conditions was systematically recorded. Written informed consent was obtained from each individual before participation in the study. Participants were subsequently divided into two groups: patients diagnosed with age-related macular degeneration (AMD) and control subjects. The diagnosis of AMD was established by an ophthalmologist. The detailed inclusion and exclusion criteria applied to both AMD patients and the control group are presented in Table 2.

### 4.2. Response of Exudative AMD to Anti-VEGF Injection Therapy

Based on clinical optical coherence tomography (OCT) findings and best-corrected visual acuity (BCVA) measurements, patients with exudative AMD were divided into two categories, namely responders and non-responders, using the same criteria as in our previous study [46], which are in line with the definitions of treatment response proposed by Amoaku et al. [11].

The effectiveness of anti-VEGF therapy (ranibizumab or bevacizumab) was assessed in patients with exudative AMD presenting with exudative or hemorrhagic changes in the macula. Only treatment-naïve patients who had not previously received intravitreal anti-VEGF injections or other treatments for neovascular AMD were included. All patients received a loading phase of three consecutive monthly intravitreal injections of ranibizumab (0.5 mg) or bevacizumab (1.25 mg), followed by a treat-and-extend regimen. Treatment intervals were extended by 2 weeks, up to a maximum of 12 weeks, in the absence of disease activity, and shortened by 2 weeks when signs of disease recurrence or progression were detected. Disease activity was defined by new or increased intraretinal or subretinal fluid on OCT, an increase in central macular thickness (CMT) associated with exudative changes, a decrease in BCVA attributable to AMD activity, or new macular haemorrhage. The anti-VEGF agent administered is shown in Table 1; the number of injections received during the first six months was not available for analysis. All patients were scheduled for a six-month follow-up after the first anti-VEGF injection. However, one patient did not attend the six-month visit and was therefore excluded from the treatment-response analysis. CMT and BCVA were assessed at baseline and six months after the initial intravitreal injection. OCT was performed with a Triton swept-source OCT device (Topcon Corporation, Tokyo, Japan; software version 10.13). CMT was defined as the mean retinal thickness in the central 1 mm subfield of the ETDRS grid and was measured automatically by the device software and checked for segmentation errors by an experienced grader masked to microbiome results. BCVA was measured using decimal visual acuity charts.

Visual acuity was evaluated at baseline and at the six-month follow-up. A decline in visual acuity was defined as a loss of more than 0.1 logMAR (one chart line, equivalent to five ETDRS letters). Changes in BCVA during the treatment period were calculated as the difference between BCVA at six months and BCVA measured before treatment.

A good treatment response was defined as the disappearance of retinal fluid on OCT six months after the first injection and/or an improvement in visual acuity of more than 0.1 logMAR. Decimal visual acuity was converted to logMAR for analysis (logMAR = −log10 of decimal acuity); a change of 0.1 logMAR corresponds to one chart line or five ETDRS letters.

A non-response was defined as an increase in CMT of at least 100 μm due to intraretinal fluid (IRF) and/or subretinal fluid (SRF), worsening haemorrhage compared with baseline, and/or a loss of more than 0.1 logMAR in visual acuity relative to baseline or the best recorded BCVA during follow-up. Changes in CMT were calculated as the difference between baseline CMT and CMT measured six months after treatment.

Because the response definitions are not mutually exclusive, non-response criteria took precedence: a patient fulfilling any non-response criterion was classified as a non-responder irrespective of fluid resolution. Patients who fulfilled neither definition (persistent but stable fluid and a BCVA change of ≤0.1 logMAR) were to be classified as responders; no patient fell into this intermediate category. Response was graded by an ophthalmologist masked to the microbiome data.

**Collection and Preparation of Study Samples**

Nasopharyngeal swab samples were collected before the start of anti-VEGF therapy for microbiota analysis according to a standardised procedure. Swabs were collected by a biotechnologist before any intravitreal treatment or eye drops on the day of sampling. Participants were asked not to eat, drink, smoke, brush their teeth or use nasal sprays for at least 4 h before sampling. The flocked swab was inserted through one nostril along the nasal floor to the posterior nasopharyngeal wall, rotated five to eight times and withdrawn. Samples were obtained using sterile FLOQSwab collection devices (COPAN Italia S.p.A., Brescia, Italy) and placed into 1 mL eNAT transport and preservation tubes containing a stabilising buffer designed to preserve nucleic acid integrity and inhibit microbial DNA degradation. Samples were transferred to the laboratory within a few minutes, as sample collection and laboratory processing were performed at the same clinical facility. Upon arrival, samples were immediately frozen at −80 °C until DNA extraction, strictly avoiding repeated freeze–thaw cycles to prevent microbial DNA fragmentation and potential alterations in microbiota composition.

**Nucleic Acid Extraction**

For nasopharyngeal microbiota analysis, nucleic acids were extracted from nasopharyngeal secretion (swab) samples using the Invitrogen PureLink Microbiome DNA Purification Kit (Invitrogen, Thermo Fisher Scientific, Carlsbad, CA, USA) according to the manufacturer’s protocol and recommendations. Microorganisms were lysed using combined thermal, chemical, and mechanical disruption with specialised glass beads. Following lysis, samples were transferred to PureLink™ spin columns, where DNA was bound, washed, and eluted in an elution buffer.

**Preparation of 16S rRNA Gene V3–V4 Region Sequencing Libraries**

A total of 64 nasopharyngeal samples were included in the microbiota analysis. To monitor potential contamination, 9 negative controls were included: 3 PCR water controls (NQC1/2/3) to assess possible contamination of PCR reagents and the working environment; 3 negative extraction controls (NQCextraction1/2/3) to evaluate contamination associated with DNA extraction kits and procedures; and 3 negative swab controls (NQCswab1/2/3) to assess contamination related to sampling devices and transport conditions.

Library preparation was performed using two 96-well plates, with PCR amplification carried out in two technical replicates per sample. The products of the two technical PCR replicates were combined, and the indexed amplicons were subsequently pooled into a single library prior to purification. Technical replicates were not sequenced or analysed separately.

For each PCR reaction, a Master Mix was prepared consisting of:

- ultra-pure laboratory-grade water,
- 5× SuperFi™ GC Enhancer (Invitrogen, Thermo Fisher Scientific Baltics UAB, Vilnius, Lithuania),
- 2× Platinum SuperFi PCR Master Mix (Invitrogen, Thermo Fisher Scientific Baltics UAB, Vilnius, Lithuania).

**Primer Indexing**

Extracted DNA was amplified using dual-index PCR with a specific primer pair targeting the V3–V4 region of the 16S rRNA gene: Forward 341F (5′-CCTACGGGNGGCWGCAG-3′) and Reverse 805R (5′-GACTACHVGGGTATCTAATCC-3′) [48].

**PCR principles**

Reaction Composition is presented in Table 3. PCR amplification was performed using a Veriti Thermal Cycler (96-well Fast) (Applied Biosystems, Thermo Fisher Scientific, Carlsbad, CA, USA). Cycling conditions are presented in Table 4.

**PCR Product Purification**

Following PCR amplification, the indexed PCR products were assessed by agarose gel electrophoresis, pooled into a single tube, and purified using the PureLink™ Quick Gel Extraction and PCR Purification Combo Kit (Invitrogen, Thermo Fisher Scientific Baltics UAB, Vilnius, Lithuania) according to the manufacturer’s instructions. PCR products were bound to spin columns using binding buffer (B2), impurities were removed using ethanol-containing wash buffer (W2), and DNA was eluted using elution buffer (E1).

**Assessment of 16S rRNA Library Size and Concentration**

Fragment size distribution and quality of the prepared 16S rRNA sequencing libraries were evaluated using the Agilent TapeStation system (Agilent Technologies, Santa Clara, CA, USA). TapeStation analysis provided fragment profiles reflecting library size distribution and confirmed their suitability for downstream sequencing. This automated preparation and analysis workflow ensured library uniformity, improved data reliability, and reduced the risk of manual processing errors.

Library concentration was determined using quantitative real-time PCR (qRT-PCR) with the Collibri Library Quantification Kit (Thermo Fisher Scientific Baltics UAB, Vilnius, Lithuania) and the QuantStudio 5 Real-Time PCR system (Applied Biosystems, Thermo Fisher Scientific, Waltham, MA, USA), enabling accurate quantification of amplifiable, sequencing-ready fragments. The resulting concentration measurement was used to calculate the dilution required for sequencing of the pooled library.

**16S rRNA Library Preparation and Sequencing Using the Illumina MiSeq Platform**

The 16S rRNA libraries were prepared for sequencing according to the manufacturer’s recommended Illumina MiSeq protocol. Libraries were denatured with sodium hydroxide to generate single-stranded DNA suitable for initiating sequencing. After denaturation, libraries were diluted in an appropriate buffer to ensure optimal sequencing conditions on the MiSeq platform.

The final sequencing mixture was prepared by combining the pooled sequencing library with sequencing controls to monitor sequencing quality.

Prepared 16S rRNA gene libraries were sequenced on the Illumina MiSeq platform using the MiSeq Reagent Kit v3 (2 × 300 bp) (Illumina, San Diego, CA, USA) according to the manufacturer’s instructions.

**Bioinformatic and statistical analysis**

### 4.3. Microbiome Data Processing

#### Sequence Denoising and ASV Inference

Raw paired-end 16S rRNA gene sequencing reads were processed using QIIME 2 (amplicon-2024.10) [49]. Sequence denoising was performed with the DADA2 algorithm [50] implemented in QIIME 2, which performs quality filtering, error correction, chimaera removal, and inference of amplicon sequence variants (ASVs).

Based on inspection of per-base quality score profiles, forward and reverse reads were truncated at 260 bp and 230 bp, respectively (trimLeft = 0/0; truncLen = 260/230). Reads with expected errors greater than two were removed (maxEE = 2/2). ASVs were inferred using the DADA2 error-correction model, and chimeric sequences were removed using the consensus method. The resulting ASV table was used for downstream taxonomic classification and diversity analyses.

**Taxonomic classification**

Taxonomic assignment was performed using a naïve Bayes classifier trained on the SILVA 138 reference database [51] trimmed to the amplified 16S rRNA gene region, implemented in the q2-feature-classifier plugin of QIIME 2 [52].

Prior to downstream analyses, non-bacterial sequences, including mitochondrial sequences, chloroplast sequences, and unassigned reads, were removed from the feature table.

Negative controls, including extraction blanks and PCR controls, were sequenced together with study samples to monitor potential contamination. These controls were inspected during quality assessment and were excluded from downstream analyses. Because low-biomass samples are susceptible to reagent and laboratory contamination [53], potential contaminant ASVs were identified using the prevalence-based approach of decontam (a one-sided Fisher’s exact test for higher prevalence in the nine negative controls than in participant samples, with a score threshold of 0.5) and removed before downstream analyses [54]. In total, 87 ASVs (2.2% of reads) were classified as contaminants; they are listed in Supplementary Table S1.

**Rarefaction and sequencing depth control**

To account for variability in sequencing depth across samples, rarefaction analysis was performed [55]. Rarefaction curves demonstrated that diversity metrics approached saturation between 6000 and 8000 reads, indicating sufficient sampling coverage for most samples. Based on these observations, a rarefaction threshold of 12,000 reads per sample was selected for downstream diversity analyses to minimise undersampling bias while retaining the majority of samples. To assess the influence of the random rarefaction draw and of the depth threshold, key analyses were repeated across 20 independent rarefactions and at a depth of 9000 reads.

Samples with sequencing depth below this threshold were excluded from diversity analyses.

### 4.4. Diversity Analyses

#### 4.4.1. Alpha Diversity

Within-sample microbial diversity was assessed using the Shannon diversity index, Observed ASV richness, and Faith’s phylogenetic diversity [56]. Group comparisons between AMD patients and controls, and between responders and non-responders, were performed using the Kruskal–Wallis test.

#### 4.4.2. Beta Diversity

Between-sample microbial community differences were evaluated using Bray–Curtis dissimilarity, normalised weighted UniFrac distance, and unweighted UniFrac distance [57]. Microbial community structure was visualised using principal coordinates analysis (PCoA).

Differences in microbial community composition between groups were assessed using PERMANOVA with 999 permutations [58]. Because PERMANOVA can be sensitive to differences in within-group variability, particularly with unequal group sizes [33], homogeneity of multivariate dispersion was evaluated using PERMDISP [59].

**Covariate-adjusted microbiome analysis**

To account for potential confounding effects of demographic and clinical variables, PERMANOVA models included age, sex and statin use as covariates and were tested with marginal (partial) tests, permuting residuals under the reduced model (equivalent to adonis2 with by = “margin” in the vegan package), so that the R^2^ and *p*-value reported for group or response are adjusted for the other terms in the model; the R^2^ of the full model is reported separately [60].

For case–control comparisons, microbial community variation was modelled as a function of disease status while adjusting for potential confounders using the model:

distance ~ Group + Age + Sex + Statin

Treatment-response analyses were performed within the AMD cohort using the model:

distance ~ Response + Age + Sex + Statin

Only samples with complete metadata were included in adjusted analyses.

In sensitivity analyses, baseline BCVA, baseline CMT and the anti-VEGF agent were added one at a time to the treatment-response model; because of the small number of non-responders, these covariates were not entered simultaneously to avoid overfitting. To assess the influence of individual patients, the response analyses were repeated after removing each non-responder in turn (leave-one-out analysis).

### 4.5. Differential Abundance Analysis

Genus-level differential abundance analysis was performed on centred log-ratio (CLR)-transformed genus counts (pseudocount 0.5), comparing groups with the Mann–Whitney U test [61] for both the case–control and the responder versus non-responder comparisons. Prior to analysis, taxa present in fewer than 10% of samples were removed to reduce sparsity and improve statistical robustness; to verify that this threshold did not remove informative low-abundance taxa, the analyses were repeated with a 5% prevalence threshold.

Multiple testing correction was performed using the Benjamini–Hochberg false discovery rate (FDR) procedure [62], and taxa with FDR-adjusted q-values < 0.05 were considered statistically significant.

### 4.6. General Statistical Considerations

All statistical tests were two-sided, and statistical significance was defined as *p* < 0.05. False discovery rate correction was applied where appropriate to account for multiple comparisons. The association between *Corynebacterium* and treatment response was examined post hoc using the Wilcoxon rank-sum test, and effect size was expressed as Cliff’s delta with a bootstrap 95% confidence interval [63]. Because this was designed as a pilot study, no a priori sample size calculation was performed; instead, the observed effect sizes were used to estimate, with Noether’s method, the sample size required for a confirmatory study [42]. Continuous clinical variables were compared using the Mann–Whitney U test and categorical variables using Fisher’s exact test.

Microbiome processing was performed using QIIME 2 (amplicon-2024.10). Taxonomic filtering, decontamination, rarefaction and all statistical analyses were performed in Python (version 3.13) with custom scripts based on NumPy, SciPy [64] and pandas; the analysis code is available from the corresponding author on request.

## 5. Conclusions

In this study, neovascular AMD status was not associated with statistically significant differences in overall nasopharyngeal microbial diversity or community composition. In contrast, baseline microbial community composition differed between patients who subsequently responded and did not respond to anti-VEGF therapy. Higher relative abundance of *Corynebacterium* was observed in non-responders in an exploratory analysis. These preliminary findings require confirmation in larger, independent cohorts before any predictive value can be assessed.

## Figures and Tables

**Table 1:** Demographic and clinical characteristics of the participants included in the analyses.

Characteristic	Controls (*n* = 30)	AMD (*n* = 25) ^d^	*p*	Responders (*n* = 19)	Non-Responders (*n* = 5) ^b^	*p*
Age, years	69.5 (58–76)	80 (73–84)	0.001	80 (74–84)	73 (68–88)	0.83
Female sex, *n* (%)	24 (80)	19 (76)	0.75	14 (74)	4 (80)	1.00
Statin use, *n* (%) ^a^	6 (20)	16 (67)	<0.001	11 (61)	4 (80)	0.62
BCVA, logMAR ^c^	—	0.40 (0.40–0.85)	—	0.40 (0.38–0.93)	0.40 (0.22–0.70)	0.39
CMT, µm	—	381 (327–438)	—	385 (338–448)	321 (302–412)	0.18
Anti-VEGF agent: ranibizumab/bevacizumab, *n*	—	21/4	—	15/4	5/0	0.54

Baseline (pretreatment) values. Continuous variables are median (IQR) and were compared with the Mann–Whitney U test; categorical variables are *n* (%) and were compared with Fisher’s exact test. ^a^ Statin use is unknown for one patient with AMD (responder). ^b^ Continuous variables for non-responders are median (range) because of the small group size. ^c^ Converted from decimal visual acuity (logMAR = −log10[decimal acuity]); higher values indicate worse vision. Visual acuity and CMT were not systematically recorded in controls. BCVA, best-corrected visual acuity; CMT, central macular thickness. ^d^ Includes one patient who did not attend the six-month visit and therefore could not be classified by treatment response (responders + non-responders = 24).

**Table 2:** Inclusion and exclusion criteria, as applied in our previous study [46].

Group	Inclusion Criteria	Exclusion Criteria
Exudative AMD	Treatment-naïve patients with exudative (neovascular) AMD, i.e., late AMD according to the AREDS classification, who provided written informed consent to participate in the study; Male and female participants aged between 50 and 99 years; Diagnosis of exudative AMD was established based on findings from optical coherence tomography (OCT); Fluorescein angiography performed when necessary to support the diagnosis; Classification of AMD is determined according to the system proposed by the Age-Related Eye Disease Study Research Group [47].	Presence of ocular conditions unrelated to AMD, including high refractive error, corneal opacity, lens opacities (nuclear, cortical, or posterior subcapsular cataract) except for mild opacities, keratitis, acute or chronic uveitis, glaucoma, or optic nerve disorders; Presence of systemic diseases such as diabetes mellitus, malignant tumours, systemic connective tissue diseases, chronic infectious and non-infectious diseases, coronary artery disease, stroke, or a history of organ or tissue transplantation; Colour fundus photographs that could not be evaluated due to obscuration of the ocular optical media or inadequate image quality. Current use of antiepileptic or sedative medications.
Control	Ophthalmologically healthy individuals; Male and female participants aged 18 years or older; No history of chronic infectious or noninfectious diseases; Provision of written informed consent for participation in the study.	Any ocular pathology or optical coherence tomography (OCT) images that could not be assessed due to opacity of the ocular optical media; Systemic disorders, including diabetes mellitus, malignant tumours, systemic connective tissue diseases, chronic infectious and non-infectious diseases, hypertension, coronary artery disease, stroke, or a history of organ or tissue transplantation; Current use of antiepileptic or sedative medications.

**Table 3:** PCR reagents and volume.

PCR Reagent	PCR Reaction Volume
Master Mix	18 µL
DNA sample	5 µL
Forward primer, Reverse primer	2 µL 2 µL
Total	27 µL

**Table 4:** PCR conditions.

PCR Cycling	PCR Cycling Conditions
Initial denaturation	98 °C 30 s.
33 cycles	
Denaturation	98 °C for 9 s
Primer annealing	50 °C for 1 min
Elongation	72 °C for 20 s
Final elongation to ensure complete synthesis of amplified fragments	72 °C for 10 min

## Data Availability

The data supporting the findings of this study are available from the corresponding author upon reasonable request, subject to applicable ethical and data protection requirements. The analysis code is available from the corresponding author on request.

## Supplementary Materials

The following supporting information can be downloaded at: https://www.mdpi.com/article/10.3390/ijms27219673/s1.
