# Supplementary material for: Nasopharyngeal Microbial Signatures Associated with Anti-VEGF Therapeutic Outcomes in Exudative Age-Related Macular Degeneration: A Pilot Study
Source: Int J Mol Sci. 2026 Oct 28;27(21):9673. doi: 10.3390/ijms27219673 (PMC13762646; doi:10.3390/ijms27219673)
Supplement: Supplementary file 1 [file ijms-27-09673-s001.zip › ijms-4646645-supplementary.pdf]

Supplementary Material

Nasopharyngeal Microbial Signatures Associated with Anti-VEGF Therapeutic Outcomes in Exudative Age-Related Macular Degeneration: A Pilot Study

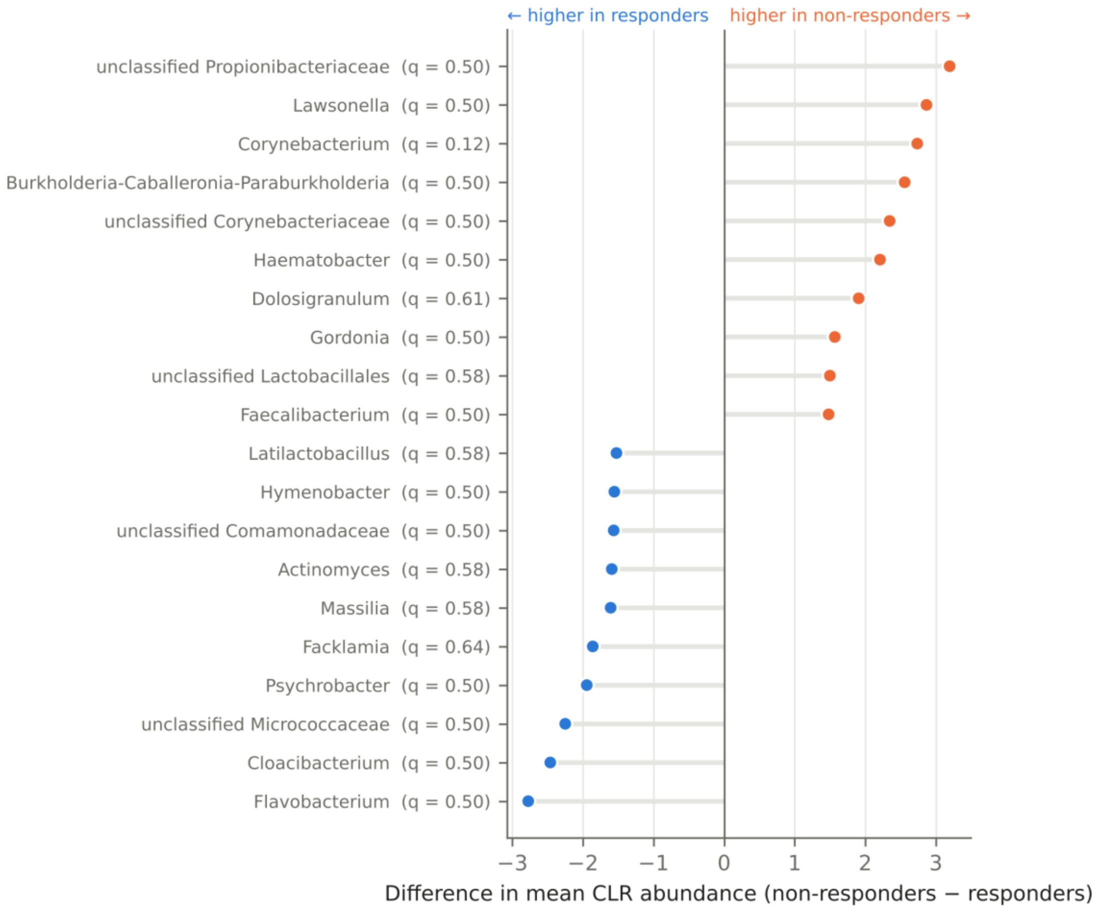

Supplementary Figure S1. Genus-level differential abundance between non-responders and responders to anti-VEGF therapy. The 20 genera with the largest estimated effect sizes are shown; FDR-adjusted q-values are given next to each genus. No genus remained statistically significant after false discovery rate correction.

Supplementary Table S1. Amplicon sequence variants (ASVs) identified as potential contaminants (prevalence method, threshold 0.5) and removed before analysis (n = 87). Prevalence is given among the 64 participant samples and the 9 negative controls (3 extraction, 3 swab and 3 PCR blanks).

ASV (first 12 characters)	Genus	Prevalence in samples (/64)	Prevalence in negative controls (/9)	Reads in samples	Reads in negative controls	Score
ba2952f339b7…	Staphylococcus	4	2	10396	159	0.156
880a712ae317…	Staphylococcus	6	3	8669	123	0.076
5614ed09636e…	Staphylococcus	4	2	3542	108	0.156
fe8794f2fc28…	unclassified Corynebacteriaceae	4	1	1898	42	0.492
80256b9e41fa…	Streptococcus	5	3	1883	279	0.054
51354f330ace…	Cutibacterium	5	3	1835	231	0.054
0332dc28bbe8…	unclassified Corynebacteriaceae	4	1	1780	42	0.492
54604027d065…	Gemella	3	1	1597	82	0.416
6bff534d2b2a…	Cutibacterium	4	2	1359	177	0.156
95ca961525db…	Cutibacterium	5	3	1124	262	0.054
6305b08357a2…	Escherichia-Shigella	1	1	665	48	0.233
fa88181f1993…	Streptococcus	3	1	612	61	0.416
e4bc1a64f9ae…	Streptococcus	3	2	573	148	0.112
195461078457…	Burkholderia-Caballeronia-Paraburkholderia	4	3	502	2279	0.036
6af2be763b58…	Burkholderia-Caballeronia-Paraburkholderia	4	2	472	1155	0.156
397e989defb6…	Corynebacterium	4	2	463	479	0.156
68f88bd13d02…	Allorhizobium-Neorhizobium-Pararhizobium-Rhizobium	4	1	440	101	0.492
6f9c4e1f1a2c…	Micrococcus	3	1	374	95	0.416
9d7452553045…	uncultured	3	2	326	245	0.112
5c02a9757544…	Burkholderia-Caballeronia-Paraburkholderia	4	3	319	1817	0.036
25b93756dca4…	Corynebacterium	3	1	313	137	0.416
5469b911f8cd…	Burkholderia-Caballeronia-Paraburkholderia	4	3	297	1412	0.036
f1643da54d1c…	Burkholderia-Caballeronia-Paraburkholderia	4	3	290	1493	0.036
721653927649…	Enhydrobacter	2	1	250	112	0.330
0d6a0de6617c…	Burkholderia-Caballeronia-Paraburkholderia	4	3	250	1362	0.036
bc2db28e83d9…	Burkholderia-Caballeronia-Paraburkholderia	3	2	239	728	0.112
1b74b7834f34…	uncultured	3	1	235	43	0.416
ebfe614fefe5…	Lactobacillus	1	2	233	158	0.038
33fec7298cf6…	Burkholderia-Caballeronia-Paraburkholderia	4	2	232	699	0.156
dcaa6cd45e8c…	uncultured	2	1	226	77	0.330
5ea53fd3fb66…	Kocuria	2	1	225	66	0.330
316fe4818238…	Corynebacterium	3	2	195	178	0.112
3766b900afd9…	Lactobacillus	2	1	191	85	0.330
f4925a8db367…	Ezakiella	2	1	186	40	0.330

ASV (first 12 characters)	Genus	Prevalence in samples (/64)	Prevalence in negative controls (/9)	Reads in samples	Reads in negative controls	Score
f73b167e4963…	Micrococcus	3	1	159	27	0.416
0a26011f281c…	Cutibacterium	2	1	145	19	0.330
0a3b9db09547…	Fusobacterium	2	1	142	74	0.330
15865a6afd7c…	Anaerococcus	3	1	141	79	0.416
e7428ce359d6…	Burkholderia-Caballeronia-Paraburkholderia	2	3	137	1066	0.012
d09eeb283cce…	Haemophilus	3	1	135	23	0.416
fba2b0174811…	Cloacibacterium	2	1	134	89	0.330
23d9f6dd7d3e…	Corynebacterium	1	3	130	255	0.005
6cbb6089d099…	Allorhizobium-Neorhizobium-Pararhizobium-Rhizobium	1	3	128	339	0.005
785a681af35d…	Cloacibacterium	2	1	124	62	0.330
32f945cc3353…	Georgenia	2	1	124	80	0.330
f7d0cb8d0f49…	Allorhizobium-Neorhizobium-Pararhizobium-Rhizobium	3	3	123	217	0.022
bcf84e1855bb…	Brevibacillus	2	1	120	18	0.330
76733b3caba7…	Brevibacillus	2	1	110	65	0.330
86f970669264…	Lactococcus	1	1	108	28	0.233
23b7f5c63523…	Corynebacterium	2	3	95	250	0.012
8f66b70386b2…	Allorhizobium-Neorhizobium-Pararhizobium-Rhizobium	1	2	94	161	0.038
1bef49b3166e…	Lactococcus	2	1	94	52	0.330
dd3e790be9d3…	Corynebacterium	1	1	85	77	0.233
5fbd69c278ca…	Corynebacterium	2	2	79	76	0.072
36d8876b345b…	Veillonella	3	1	76	74	0.416
b2460b045f61…	Latilactobacillus	1	1	75	81	0.233
bb210a27c418…	Micrococcus	1	1	73	65	0.233
8413257fa9cb…	Haemophilus	1	1	70	77	0.233
ba6dee6d8372…	unclassified Xanthobacteraceae	1	2	70	151	0.038
13f3be94edd0…	Staphylococcus	4	2	69	38	0.156
7ba67adf0e80…	Brevibacillus	1	1	69	79	0.233
d4b4f5d7d0a0…	Porphyromonas	1	1	65	39	0.233
440fd12bee6d…	Brevibacillus	1	2	63	83	0.038
a08c3d7361c5…	Cloacibacterium	2	2	62	77	0.072
79433eafc076…	unclassified Sphingomonadaceae	1	1	59	78	0.233
42a8ce65a393…	Brevibacillus	1	1	50	24	0.233
51620adb2ff1…	Staphylococcus	4	1	48	15	0.492
c575b9b706e6…	Anaerococcus	1	2	47	218	0.038
10f1db02642d…	Haemophilus	2	1	45	73	0.330
9240d07c9a0b…	Actinomyces	1	1	44	32	0.233
39137553209d…	Georgenia	1	1	41	23	0.233
51773b427d34…	Neisseria	1	1	40	55	0.233

ASV (first 12 characters)	Genus	Prevalence in samples (/64)	Prevalence in negative controls (/9)	Reads in samples	Reads in negative controls	Score
47293d36e4ec…	Corynebacterium	1	1	40	57	0.233
b072eaa68630…	unclassified Microbacteriaceae	1	1	37	69	0.233
9881b45defe8…	Alloprevotella	1	1	37	42	0.233
e2cc024c3188…	Allorhizobium-Neorhizobium-Pararhizobium-Rhizobium	1	1	36	28	0.233
7386bff83020…	Corynebacterium	1	2	29	86	0.038
322ce6e25226…	Haemophilus	2	1	27	98	0.330
9d8a4d8cbf7f…	Streptococcus	1	1	27	95	0.233
7408021b82c4…	Corynebacterium	1	1	25	53	0.233
f5dec1c9e625…	Haemophilus	1	1	25	70	0.233
b2a540e6ba2a…	SM2D12	1	1	16	51	0.233
506891ba0161…	Lawsonella	1	1	15	35	0.233
33b42f29288a…	Cloacibacterium	1	1	14	2	0.233
f3357ef323de…	Enhydrobacter	1	1	13	50	0.233
539acc71f66a…	Methylobacterium-Methylorubrum	1	1	12	105	0.233
35f5349c4883…	Brevibacillus	1	1	12	9	0.233

Supplementary Table S2. Sensitivity analyses (p-values). PERMANOVA models adjusted for age, sex and statin use (marginal tests, 999 permutations). BC, Bray–Curtis; UU and WU, unweighted and weighted (normalised) UniFrac; R/NR, responders/non-responders.

**Analysis**	**n (R/NR)**	**Shannon, AMD vs. controls**	**Adjusted PERMANOVA BC, AMD vs. controls**	**Observed ASVs, R vs. NR**	**Adjusted PERMANOVA BC, R vs. NR**	**Adjusted PERMANOVA UU, R vs. NR**	**Adjusted PERMANOVA WU, R vs. NR**	**Corynebacterium, Wilcoxon p (Cliff's δ)**
Primary analysis (12,000 reads; decontam 0.5)	55 (19/5)	0.892	0.067	0.004	0.015	0.004	0.006	0.009 (0.75)
20 repeated rarefactions (range)	55 (19/5)	0.852–0.946	0.051–0.077	0.004–0.006	0.008–0.020	0.001–0.007	0.002–0.007	0.009–0.009
Rarefaction depth 9,000 reads	60 (23/5)	0.982	0.130	0.003	0.013	0.002	0.001	0.008 (0.74)
decontam threshold 0.1	56 (20/5)	0.793	0.057	0.008	0.013	0.003	0.007	0.010 (0.74)
No decontam (taxonomic filtering only)	56 (20/5)	0.742	0.057	0.008	0.011	0.003	0.006	0.010 (0.74)
Leave-one-out: non-responder 636EDL removed	54 (19/4)	—	—	0.012	0.002	—	0.001	<0.001 (0.95)
Leave-one-out: non-responder 649EVZB removed	54 (19/4)	—	—	0.011	0.213	—	0.042	0.035 (0.68)
Leave-one-out: non-responder 650EBAG removed	54 (19/4)	—	—	0.011	0.382	—	0.050	0.035 (0.68)
Leave-one-out: non-responder 653EAV removed	54 (19/4)	—	—	0.005	0.012	—	0.027	0.027 (0.71)
Leave-one-out: non-responder 668EVC removed	54 (19/4)	—	—	0.011	0.009	—	0.002	0.027 (0.71)
Controls younger than 50 years excluded	50	0.741	0.062	—	—	—	—	—
